# Persistent Type B Hyperlactataemia as a Diagnostic Clue to Occult Diffuse Large B-cell Lymphoma-Associated Haemophagocytic Lymphohistiocytosis

**DOI:** 10.7759/cureus.116914

**Published:** 2026-09-25

**Authors:** Nasiba Hassan, Kavish Khatib

**Affiliations:** 1 Internal Medicine, Great Western Hospital, Swindon, GBR; 2 Acute Medicine, Great Western Hospital, Swindon, GBR

**Keywords:** cholestatic liver injury, diffuse large b-cell lymphoma (dlbcl), haemophagocytic lymphohistiocytosis, malignancy-associated hlh, occult lymphoma, type b hyperlactataemia

## Abstract

Haemophagocytic lymphohistiocytosis (HLH) is a rare, life-threatening hyperinflammatory disorder characterised by excessive immune activation. It may be primary or secondary, with secondary HLH associated with a range of triggers; the most common are infections, autoimmune and autoinflammatory conditions, haematological malignancies and other immune‑dysregulatory states.

We present a case of a man in his 70s who presented with a two-week history of exertional dyspnoea, profound fatigue and anorexia. Initial investigations demonstrated marked hyperferritinaemia, thrombocytopenia, mild anaemia, cholestatic liver dysfunction and disproportionately elevated lactate without acidosis. Despite intravenous fluids, lactate remained persistently elevated, consistent with type B hyperlactataemia. Computed tomography (CT) of the abdomen and pelvis demonstrated appearances suggestive of hepatitis but no organomegaly, significant lymphadenopathy or biliary obstruction. Despite empirical antibiotics and fluid resuscitation, he developed progressive pancytopenia, worsening liver dysfunction and rising lactate. Further investigations supported secondary HLH, with an HScore of 186, hypertriglyceridaemia and markedly elevated lactate dehydrogenase. Initial treatment with corticosteroids and anakinra resulted in limited response. An autoimmune trigger was initially considered given his Raynaud's phenomenon and ongoing connective tissue disease investigation. However, bone marrow biopsy subsequently confirmed diffuse large B-cell lymphoma (DLBCL). Reduced-dose rituximab, cyclophosphamide, doxorubicin, vincristine and prednisone/prednisolone (R-mini-CHOP) was commenced, resulting in the resolution of hyperlactataemia, normalisation of blood counts and liver function and improvement in performance status. PET-CT after two cycles demonstrated a complete metabolic response.

This case highlights the importance of considering secondary HLH in patients with unexplained cytopenias, hyperferritinaemia, liver dysfunction and persistent hyperlactataemia. Persistent hyperlactataemia despite adequate fluid resuscitation should prompt the consideration of systemic causes beyond tissue hypoperfusion. Bone marrow biopsy is crucial for identifying occult malignancy when conventional imaging fails to show overt malignancy. The early recognition of HLH and investigation for an underlying aetiology may facilitate timely definitive treatment.

## Introduction

Haemophagocytic lymphohistiocytosis (HLH) is a rare, life-threatening syndrome characterised by uncontrolled immune activation, hypercytokinaemia and severe systemic inflammation [1]. Although inherited forms predominantly occur in children, secondary HLH is more common in adults and may arise from a broad range of conditions, including infections, autoimmune or autoinflammatory conditions, haematological malignancies, metabolic disease, iatrogenic immunosuppression, transplantation and acquired immune deficiency [2].

Lymphomas are an important cause of malignancy-associated HLH and are associated with poor clinical outcomes. The clinical presentation may overlap with sepsis and multiorgan dysfunction, creating diagnostic uncertainty and potentially delaying the recognition of the underlying malignancy [3]. Identifying lymphoma-associated HLH can be challenging because several HLH-2024 criteria, including fever, cytopenias, hyperferritinaemia and splenomegaly, may also occur because of the underlying malignancy or its complications [4].

Hyperlactataemia is conventionally classified as type A or type B. Type A hyperlactataemia results from tissue hypoxia or hypoperfusion, such as in shock, sepsis or severe hypoxaemia. In contrast, type B hyperlactataemia occurs despite adequate tissue oxygen delivery and perfusion and may be associated with various pathologies affecting metabolic pathways, such as malignancy (increased glycolysis), hepatic dysfunction (decreased gluconeogenesis), drugs or toxins, thiamine deficiency or metabolic disorders [5]. In aggressive haematological malignancies, persistent type B hyperlactataemia may reflect increased tumour glycolysis despite adequate oxygen availability (the Warburg effect) and has been associated with poor outcomes [6]. Persistent unexplained lactate elevation despite the correction of potential hypoperfusion or other reversible causes may therefore provide a clinical clue to an underlying malignant process, although it is neither specific for malignancy nor diagnostic in isolation. However, the diagnostic significance of persistent type B hyperlactataemia in adults presenting with secondary HLH associated with previously undiagnosed lymphoma is not well described in the literature, particularly when initial imaging is unrevealing.

We report a case in which persistent type B hyperlactataemia and cholestatic jaundice were the dominant features of secondary HLH as the initial manifestation of occult diffuse large B-cell lymphoma (DLBCL). The presence of cholestatic liver dysfunction may further complicate diagnostic evaluation by initially suggesting a primary hepatobiliary disorder. This case highlights persistent unexplained hyperlactataemia as a potential additional clue to an underlying malignant process in patients with HLH while emphasising the importance of comprehensive investigation for secondary causes, including timely bone marrow examination in the presence of worsening cytopenias.

## Case presentation

Initial presentation and clinical assessment

A man in his late 70s was referred by his general practitioner to the Medical Assessment Unit with a two-week history of progressive lethargy, dry cough, exertional dyspnoea, intermittent abdominal pain with loose stools, anorexia and generalised fatigue. He reported no weight loss or night sweats before admission, and fever was first documented during his hospital stay.

His past medical history was notable for atrial flutter, previously managed with catheter ablation, glaucoma, mild tricuspid regurgitation and Raynaud's phenomenon, for which he was undergoing investigation for an underlying connective tissue disorder. His medications included edoxaban, atorvastatin, famotidine, latanoprost and diltiazem, which had recently been discontinued by the patient himself, reportedly due to worsening Raynaud's symptoms and concern that the medication might be contributing to it.

On admission, he was hypotensive and tachycardic. His initial observations were as follows: blood pressure, 99/61 mmHg; heart rate, 133 beats per minute (bpm); respiratory rate, 24 breaths/minute; temperature, 36.8°C; and oxygen saturation, 99% on room air. An electrocardiogram confirmed recurrent atrial flutter with a rapid ventricular response (Figure 1). Following initial management, his blood pressure stabilised at 126/87 mmHg, and his heart rate decreased to 90 beats/minute with digoxin.

**Figure 1:**
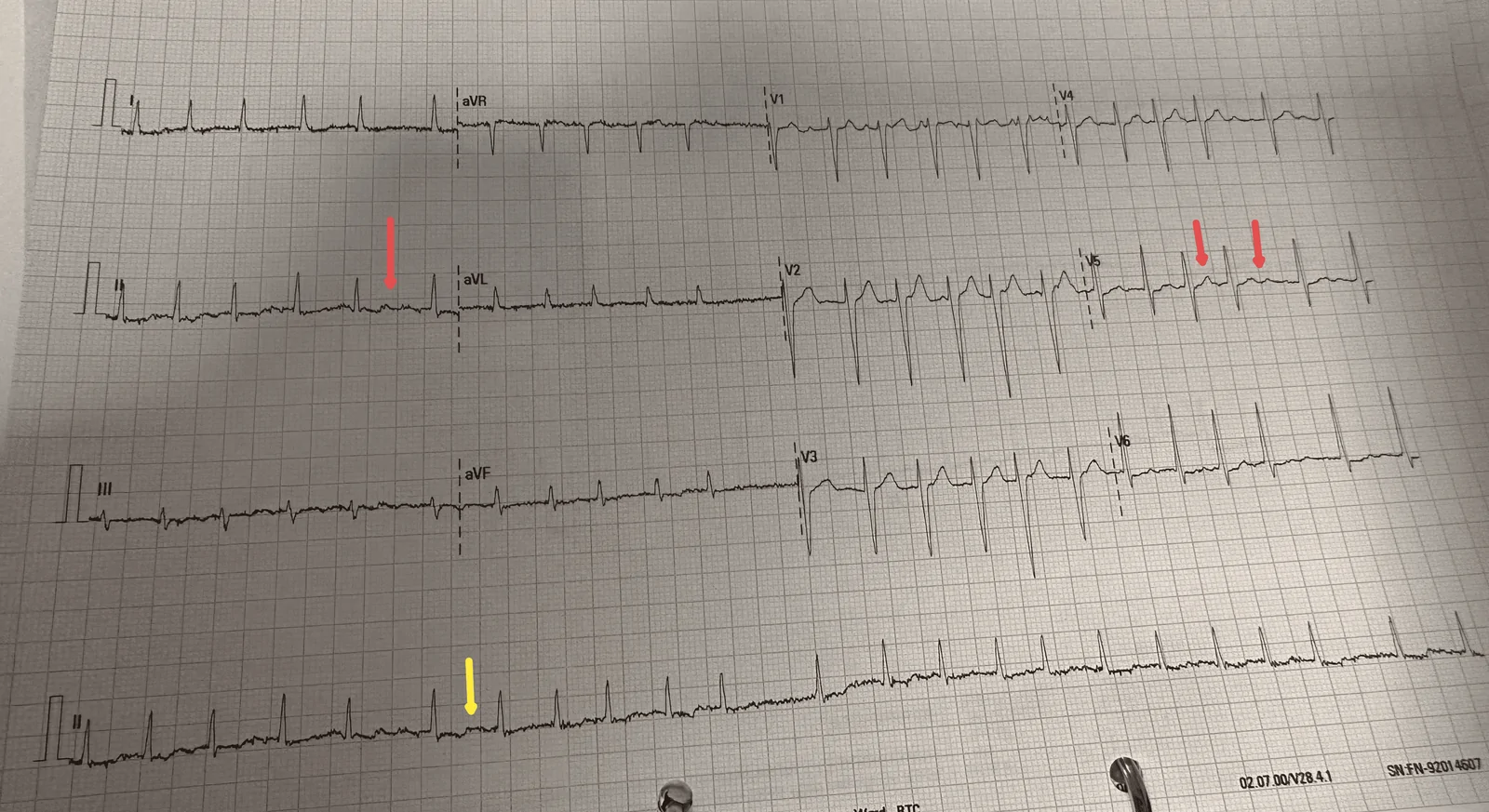
Twelve-lead electrocardiogram demonstrating atrial flutter with rapid ventricular response. The red arrows highlight the irregular R-R intervals. The yellow arrows indicate the absence of discrete P waves and the presence of a sawtooth pattern (flutter waves), consistent with atrial flutter and a rapid ventricular response (heart rate: ~134 bpm). bpm: beats per minute

Physical examination revealed no palpable peripheral lymphadenopathy, hepatomegaly or splenomegaly, and the abdomen was non-tender. The remainder of the cardiovascular, respiratory and abdominal examinations were unremarkable.

Initial blood tests demonstrated significant thrombocytopenia, mild anaemia, elevated C-reactive protein and mild hyponatremia. A blood film confirmed thrombocytopenia without other significant abnormalities. The white cell, lymphocyte, and neutrophil counts were initially within normal limits. Liver function tests demonstrated a predominantly cholestatic pattern, and ferritin was markedly elevated (Table 1). The initial differential for the abnormal liver function tests on medical review was extrahepatic biliary obstruction, portal vein thrombosis or hepatic congestion. Haematinic investigations were also performed as part of the assessment of the mild anaemia. The elevated ferritin was initially considered in the context of the inflammatory response.

**Table 1:** Baseline haematological, biochemical, liver and HLH‑associated parameters. PT, prothrombin time; aPTT, activated partial thromboplastin time; eGFR, estimated glomerular filtration rate; LDH, lactate dehydrogenase; HLH, haemophagocytic lymphohistiocytosis; AST, aspartate aminotransferase; ALT, alanine aminotransferase; ALP, alkaline phosphatase; MCV, mean corpuscular volume; MCH, mean corpuscular haemoglobin; MCHC, mean corpuscular haemoglobin concentration

Parameters	Initial values	Normal values
Haematological		
Haemoglobin	131	130-170 g/L
MCV	90	80-100 fL
MCH	30	27-33 pg
MCHC	340	320-360 g/L
Reticulocytes	59.7	40.00-120.00 10^9^/L
White cell count	4.82	3.7-11 10^9^/L
Platelet	71	150-400 10^9^/L
Lymphocyte	1.38	0.9-4.0 10^9^/L
Neutrophils	2.41	1.7-7.5 10^9^/L
Blood film	Platelets appear reduced on film; no clumping seen	-
Biochemical		
Sodium	140	133-146 mmol/L
Potassium	4.59	3.5-5.3 mmol/L
Urea	9.2	2.5-7.8 mmol/L
Creatinine	87	59-104 µmol/L
eGFR	74	>90 mL/minute/1.73 m^2^
C-reactive Protein	111	0-5 mg/L
Amylase	22	28-100 IU/L
Calcium	2.40	2.2-2.6 mmol/L
Vitamin B12	385	180-914 ng/L
Folate	13.7	3.1-20 µg/L
Liver screen		
ALT	55	<50 IU/L
ALP	293	30-130 IU/L
Bilirubin	37	0-21 µmol/L
LDH	3221	208-378 IU/L
Albumin	38	0-21 g/L
PT	14.5	8.8-12.8 seconds
PT Ratio	1.3	0.8-1.2
aPTT	26.4	19.0-28.6 seconds
aPTT ratio	1	0.8-1.2
HLH associated		
Ferritin	2199	30-300 µg/L
Triglyceride	5.05	<1.7 mmol/L
Fibrinogen	4.2	2.3-4.7 g/L
AST	156	<50 IU/L

Venous blood gas analysis performed as part of the initial assessment demonstrated a disproportionately elevated lactate level in the absence of systemic metabolic acidosis (Table 2). Lactate remained persistently elevated despite intravenous fluid resuscitation, prompting the consideration of type B hyperlactataemia. The initial lactate was 7.2 mmol/L on admission, decreasing only marginally to 6.7 mmol/L following 1 L of intravenous fluids and remaining elevated at 6.8 mmol/L over the subsequent 24 hours despite further fluid resuscitation. The lactate continued to rise throughout the admission until reduced-dose rituximab, cyclophosphamide, doxorubicin, vincristine and prednisone/prednisolone (R-mini-CHOP) was commenced.

**Table 2:** Arterial blood gas parameters before and after intravenous fluid administration. pH, potential of hydrogen; pCO₂, partial pressure of carbon dioxide; HCO₃, bicarbonate; BE, base excess

Parameters	Initial	After intravenous fluid	Normal values
PH	7.361	7.387	7.35-7.45
pCO₂	5.87	5.86	5.5-6.4 (kPa)
HCO₃	22.1	22.7	23-28 mmol/L
BE	-0.5	-1.0	+1 to -2 mmol/L
Lactate	7.2	6.7	0.5-2.2 mmol/L

Given the persistent hyperlactataemia despite initial haemodynamic stabilisation and fluid resuscitation, further investigation was undertaken to exclude ongoing tissue hypoperfusion and other potential type A causes. Computed tomography (CT) of the abdomen and pelvis was therefore performed to investigate potential occult mesenteric ischaemia or portal vein thrombosis. This demonstrated hepatic parenchymal changes consistent with hepatitis but no biliary obstruction, solid organ lesions or significant intra-abdominal lymphadenopathy, with no radiological evidence of an obvious intra-abdominal malignancy (Figure 2). In the context of persistent hyperlactataemia despite haemodynamic stabilisation and the exclusion of apparent type A causes, a type B mechanism was subsequently considered.

**Figure 2:**
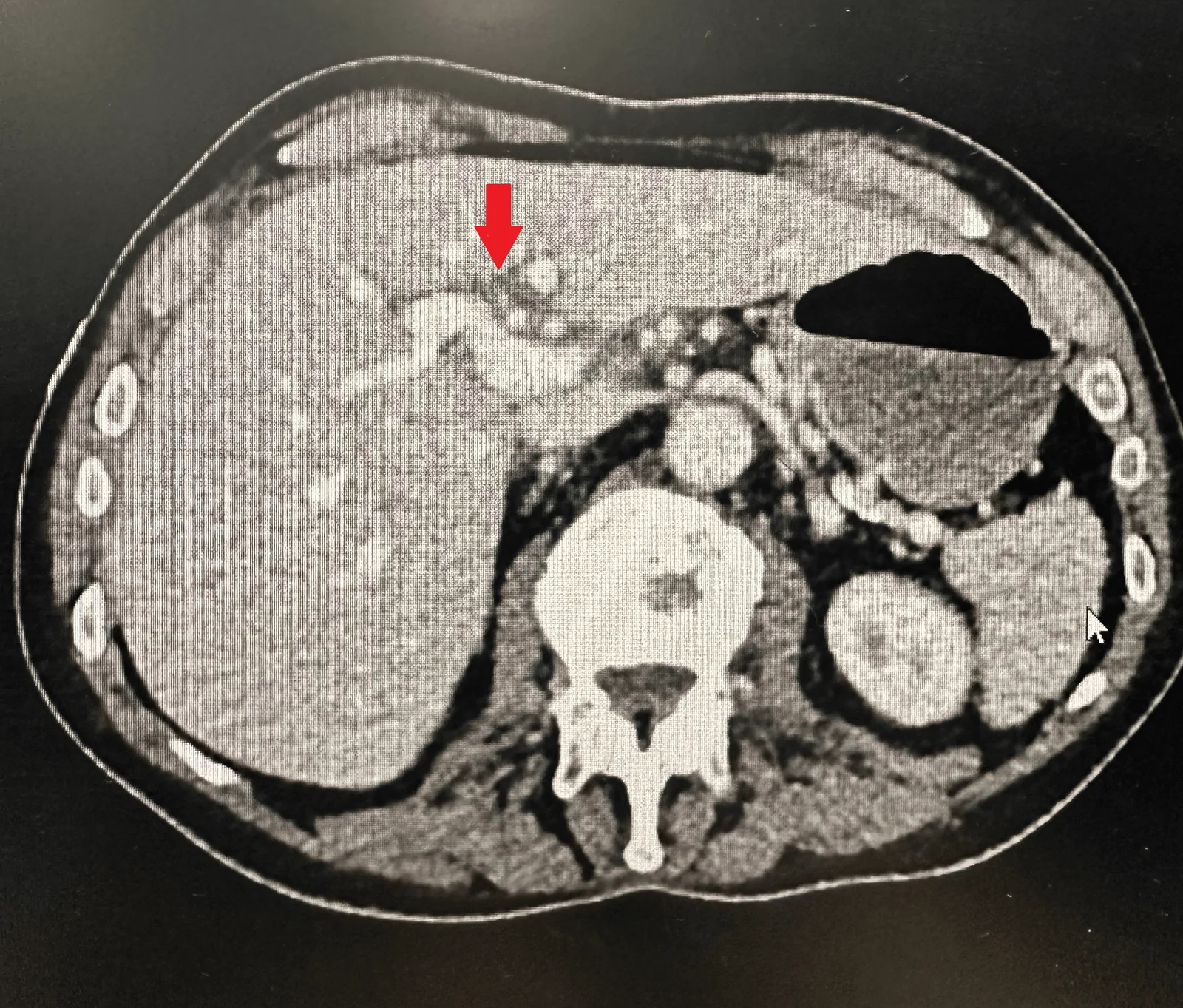
Contrast-enhanced CT of the abdomen demonstrating mild periportal oedema (arrow) and parenchymal changes consistent with hepatitis, without focal hepatic lesions or biliary obstruction. CT: computed tomography

During admission, he developed recurrent episodes of fever. Initial investigations for infection, including chest radiography and urine microscopy and culture, were unremarkable. Blood culture remained negative. Given persistent fever and hyperlactatemia, empirical intravenous antibiotics were commenced, while an infective source was sought.

Despite these measures, his clinical condition continued to deteriorate over the following days, with worsening liver dysfunction, progressive pancytopenia and persistently elevated lactate. Repeat ferritin demonstrated a further increase, raising increasing concern for an underlying hyperinflammatory process. An autoimmune screen and non-invasive liver screen were performed to investigate alternative causes of the clinical and biochemical abnormalities and were negative (Table 3). Aspartate aminotransferase (AST) also increased markedly (Table 1).

**Table 3:** Special investigations. ANA, antinuclear antibody; ANCA, antineutrophil cytoplasmic antibodies; anti-dsDNA, anti-double-stranded DNA antibody; ENA, extractable nuclear antigen; AFP, alpha-fetoprotein; anti-LKM antibody, anti-liver kidney microsomal antibody; anti-tTG antibody, anti-tissue transglutaminase antibody; IgA, immunoglobulin A; IgG, Immunoglobulin G; IgM, immunoglobulin M; CU, coeliac unit

Parameters	Values	Normal
Autoimmune screen		
ESR	6	1-30 mm/hour
ANA	Negative	-
ANCA	Negative	-
Immunoglobulins	Normal (IgG, IgA and IgM)	-
Serum protein electrophoresis	Normal	-
Serum immunofixation	No monoclonal protein	-
Anti-dsDNA	11.2	0-26.9 IU/mL
ENA panel antibodies	Negative	-
Alpha 1 anti-trypsin	256	110-210 mg/dL
Anti-LKS antibodies	Negative	
AFP	5.8	0-7 Ku/L
Anti-tTG IgA	<1.9	0-19.9 CU
Systemic sclerosis immunoblots	Negative	-
Complements	C3-1.51 and C4-0.27	C3, 0.90-1.80 g/L; C4, 0.10-0.40 g/L
Toxicology		
Salicylate level	<15	15-30 mg/dL
Ethanol level	<10	<10 mg/100 mL
Methanol level	<100	<100 mg/L

Diagnosis of HLH and initial management

The combination of persistent fever, progressive cytopenias, marked hyperferritinaemia, liver dysfunction and clinical deterioration raised the suspicion of haemophagocytic lymphohistiocytosis (HLH). Further investigations demonstrated hypertriglyceridaemia of 5.05 mmol/L and a markedly elevated lactate dehydrogenase of 3221 U/L. An HScore was calculated using contemporaneous laboratory values on day 5 of admission rather than admission blood results. The HScore was 230, supporting a high probability of HLH, and a clinical diagnosis of secondary HLH was made. The individual HScore components are summarised in Table 4.

**Table 4:** HScore components and calculation. AST: aspartate aminotransferase

HScore component	Patient value	Points
Fever	38.6	20
Ferritin	10866 µg/L	30
Triglycerides	5.05 mmol/L	20
Fibrinogen	1.2 g/L	10
AST	320 U/L	20
Organomegaly	Absent	0
Cytopaenia (≥3 lineages)	Present	30
Haemophagocytosis on marrow	Not yet confirmed	0
Total HScore	230	

Haematology was consulted and recommended a diagnostic bone marrow trephine biopsy, along with supportive management, while the underlying cause was investigated. In parallel, investigations were undertaken to identify an infectious, autoimmune or malignant trigger. A comprehensive infectious work-up was performed, with all results negative (Table 5). In addition, CT of the thorax and ultrasound of the abdomen and pelvis were performed as part of the further assessment and did not reveal any evidence of malignancy or signs suggestive of cholecystitis or gallstones.

**Table 5:** Microbiological investigations. EBV, Epstein-Barr virus; HIV, human immunodeficiency virus; COVID, coronavirus disease; RSV, respiratory syncytial virus

Tests	Results
Viral hepatitis (A, B, C, D and E) screen	Not detected
HIV	Not detected
EBV	Not detected
Blood culture	No growth
Urine microscopy	No growth
COVID, RSV and Influenza A and B	Not detected

As his clinical condition continued to deteriorate, the case was rediscussed with the haematology team. Intravenous methylprednisolone (1 g daily) was commenced for three days, together with allopurinol and intravenous fluids on the sixth day of admission. Tumour lysis parameters were monitored regularly because of the possibility of an underlying haematological malignancy.

Gastroenterology also reviewed the patient and considered the liver dysfunction most consistent with HLH‑related hepatic injury, recommending magnetic resonance cholangiopancreatography (MRCP) to exclude an underlying hepatobiliary cause.

Rheumatology subsequently initiated intravenous anakinra 300 mg daily. Following three days of pulse methylprednisolone, he was transitioned to oral prednisolone 60 mg daily. There was no significant clinical or biochemical improvement, with persistent cytopenias, liver dysfunction and hyperlactataemia. Oral prednisolone 60 mg daily was continued until the first cycle of R‑mini‑CHOP, approximately 9-10 days after starting immunomodulatory therapy. Thereafter, corticosteroids were incorporated into the R‑mini‑CHOP regimen, with prednisolone 40 mg/m² orally once daily on days 1-5 of each 21‑day cycle, in accordance with the protocol. Anakinra was discontinued around the time of the first chemotherapy cycle as the patient showed marked clinical and biochemical improvement following the first cycle of R‑mini‑CHOP.

Identification of the underlying lymphoma

Diagnostic bone marrow trephine histology subsequently confirmed diffuse large B‑cell lymphoma (DLBCL), establishing a diagnosis of lymphoma‑associated HLH after (Figures 3, 4, 5, 6). Haemophagocytosis was not reported on this sample. The histopathological findings are summarised in Table 6.

**Table 6:** Histopathological investigations. CD, cluster of differentiation; MUM1, multiple myeloma oncogene 1; TdT, terminal deoxynucleotidyl transferase; AE, antibody to cytokeratins; PGM1, phosphoglucomutase 1; c-Kit, KIT proto-oncogene receptor tyrosine kinase; MF-1, myelofibrosis grade 1; MF-2, myelofibrosis grade 2; BCL-6, B‑cell lymphoma 6

Tests	Results	Interpretation
Bone marrow trephine biopsy	Hypercellular marrow infiltrated by diffuse large B‑cell lymphoma (DLBCL), germinal centre B‑cell (GCB) type. Reticulin fibres increased (MF‑1 to MF‑2).	Confirms bone marrow involvement by DLBCL. Increased reticulin fibres, graded MF‑1 to MF‑2, indicate mild‑to‑moderate reticulin fibrosis.
Immunohistochemistry and in situ hybridisation	Neoplastic cells positive for CD20, CD79a and BCL‑6; negative for CD3, CD5, CD10, MUM1, CD30, CD23, cyclin D1, TdT, CD34, c‑KIT, CD56, CD123, AE1/AE3 and PGM1. No light‑chain restriction demonstrated by in situ hybridisation. Ki‑67 proliferation index approximately 70%.	The immunophenotype confirms B‑cell differentiation (CD20+ and CD79a+) and is consistent with a GCB phenotype according to the Hans algorithm (CD10-, BCL‑6+ and MUM1-). The negative markers help exclude alternative haematolymphoid and non-haematolymphoid diagnoses in conjunction with the morphological findings. AE1/AE3 negativity provides no evidence of epithelial differentiation. A high Ki‑67 proliferation index (70%) indicates a high proliferative fraction. The absence of demonstrable light‑chain restriction does not exclude DLBCL, as clonality may not be demonstrable by in situ hybridisation, particularly in large B‑cell lymphomas.

**Figure 3:**
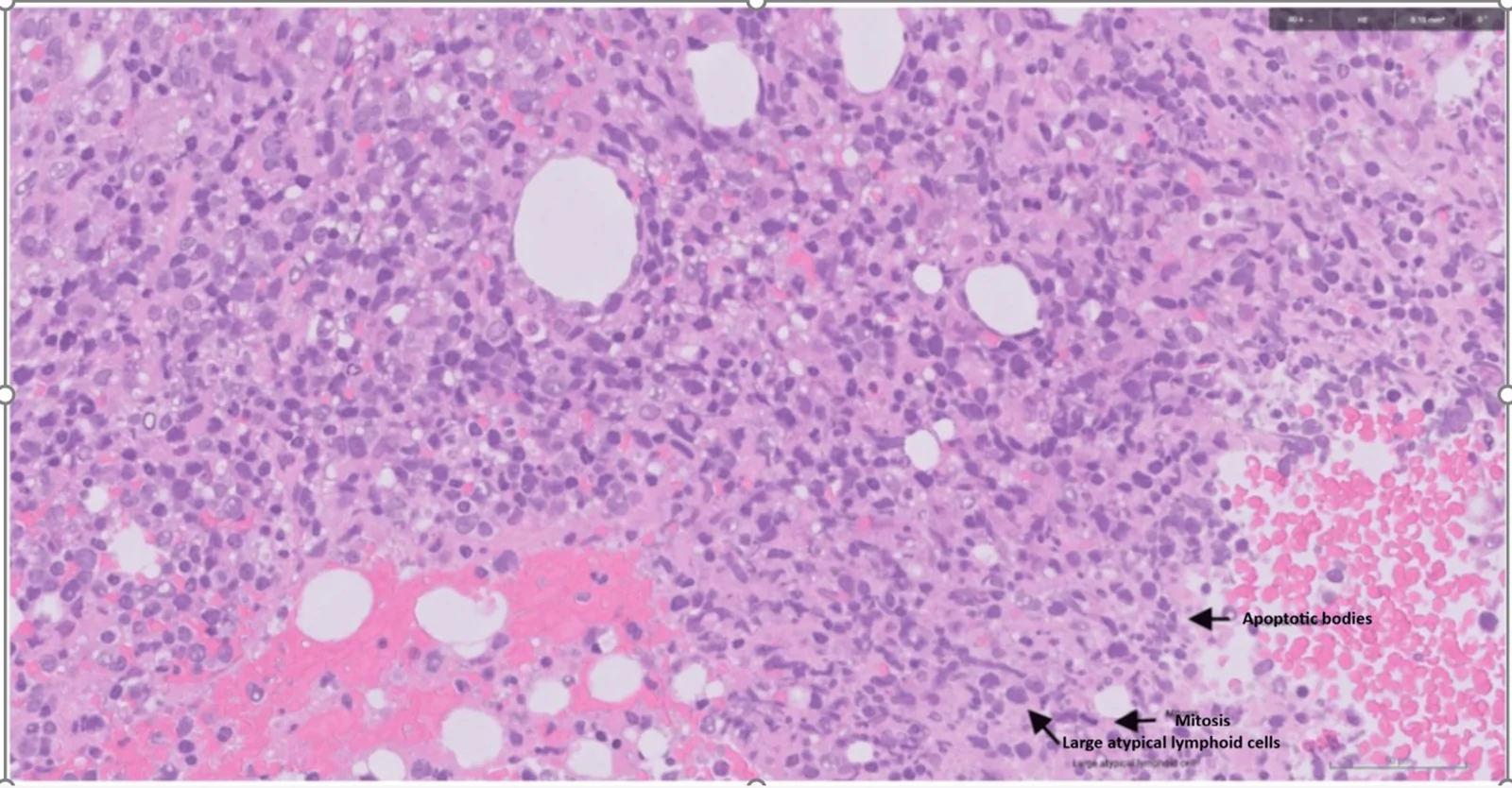
Bone marrow biopsy demonstrating diffuse large B-cell lymphoma (haematoxylin and eosin stain). Bone marrow trephine biopsy showing diffuse infiltration by large, pleomorphic atypical lymphoid cells with coarse chromatin and brisk mitotic activity. Numerous apoptotic bodies and focal crush artefact are present; representative apoptotic bodies, mitotic figures and large atypical lymphoid cells are indicated by arrows.

**Figure 4:**
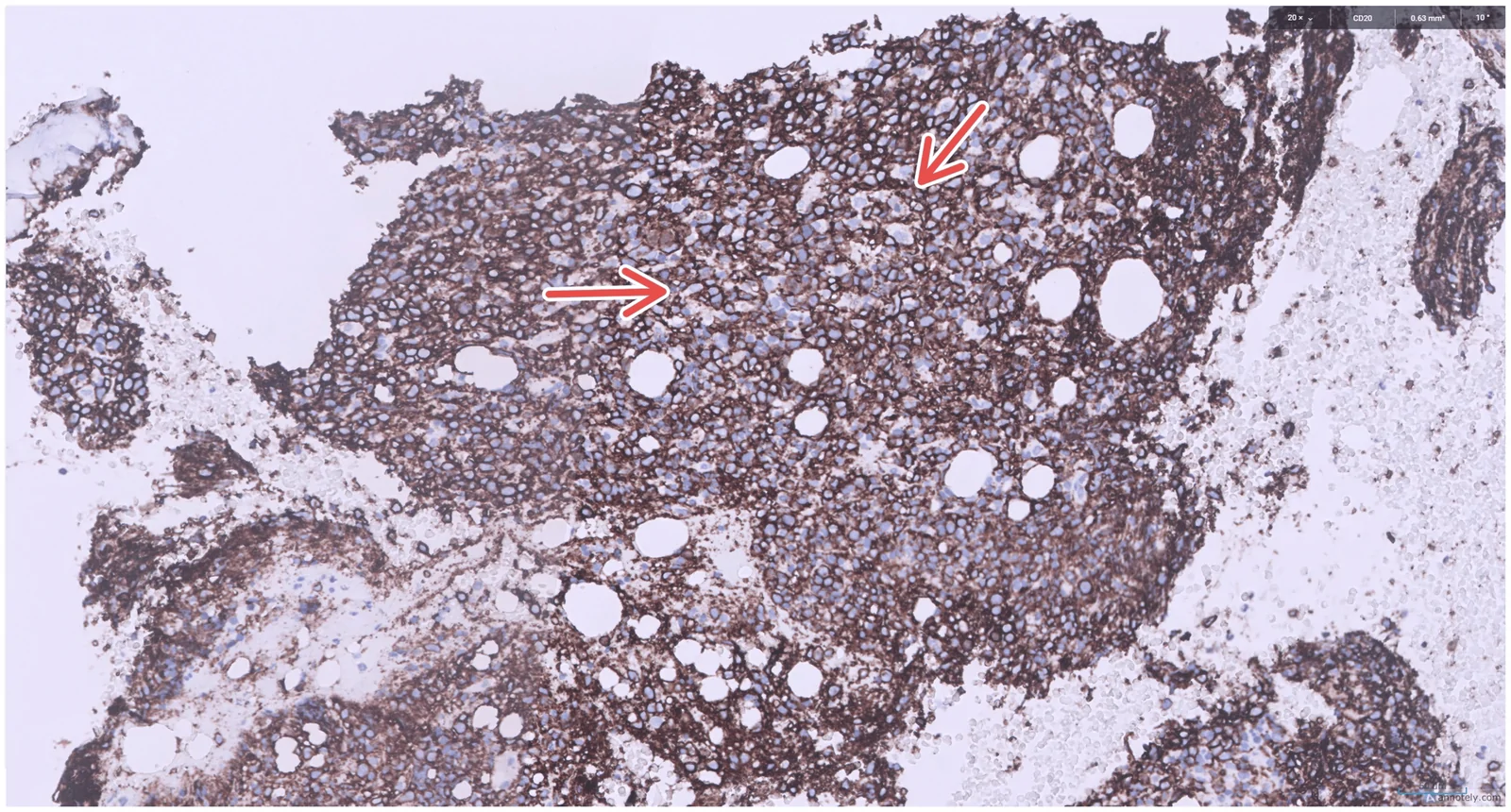
CD20 immunohistochemical staining of bone marrow trephine biopsy. Immunohistochemistry demonstrates diffuse, strong CD20 positivity within the neoplastic B‑cell infiltrate (red arrows), consistent with a B‑cell lineage. CD20: cluster of differentiation 20

**Figure 5:**
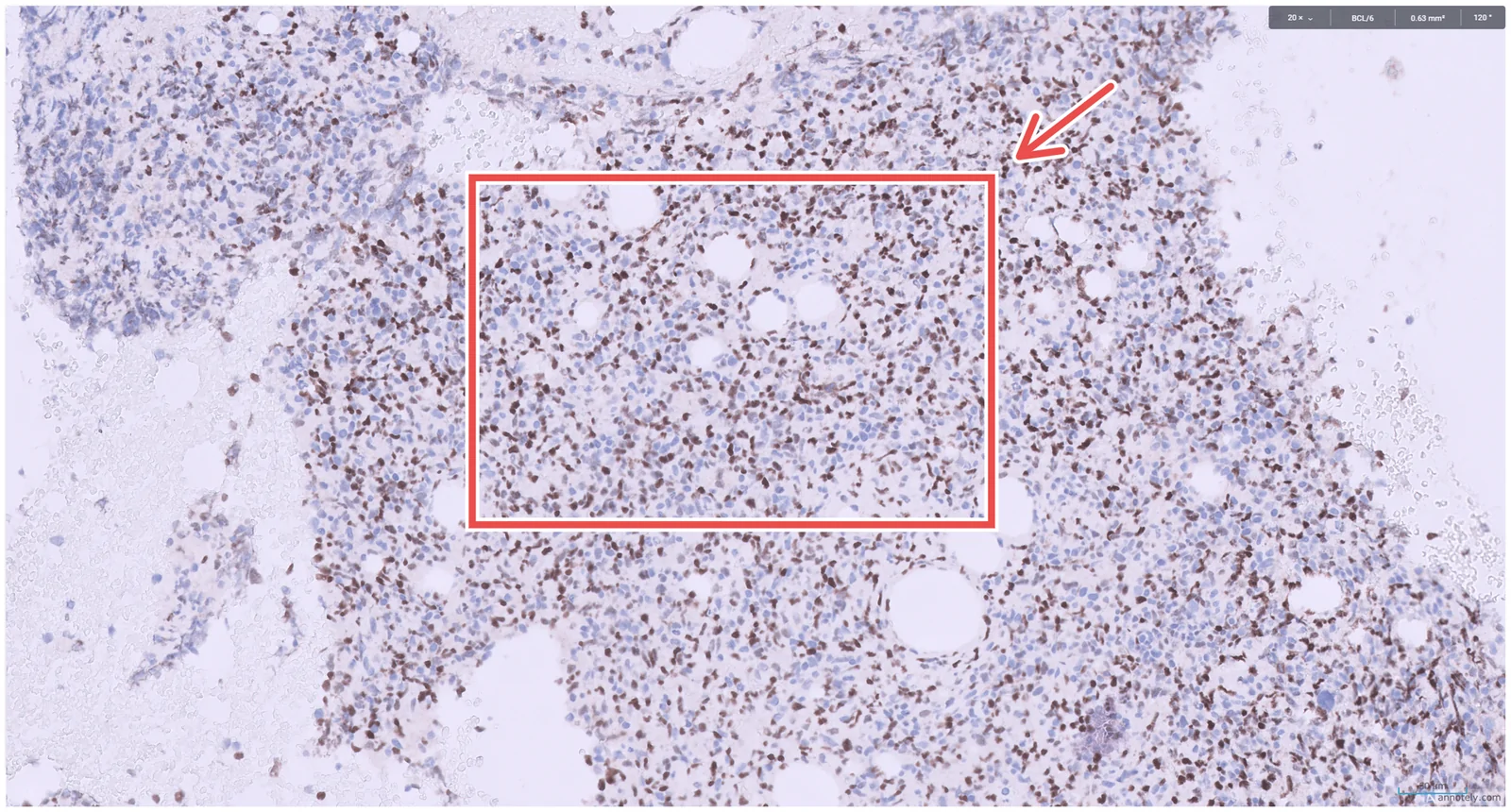
BCL‑6 immunohistochemical staining of bone marrow trephine biopsy. BCL‑6 immunohistochemistry demonstrates nuclear positivity in the neoplastic lymphoid infiltrate (red outline with arrow), supporting a germinal-centre B‑cell phenotype. BCL-6: B‑cell lymphoma 6

**Figure 6:**
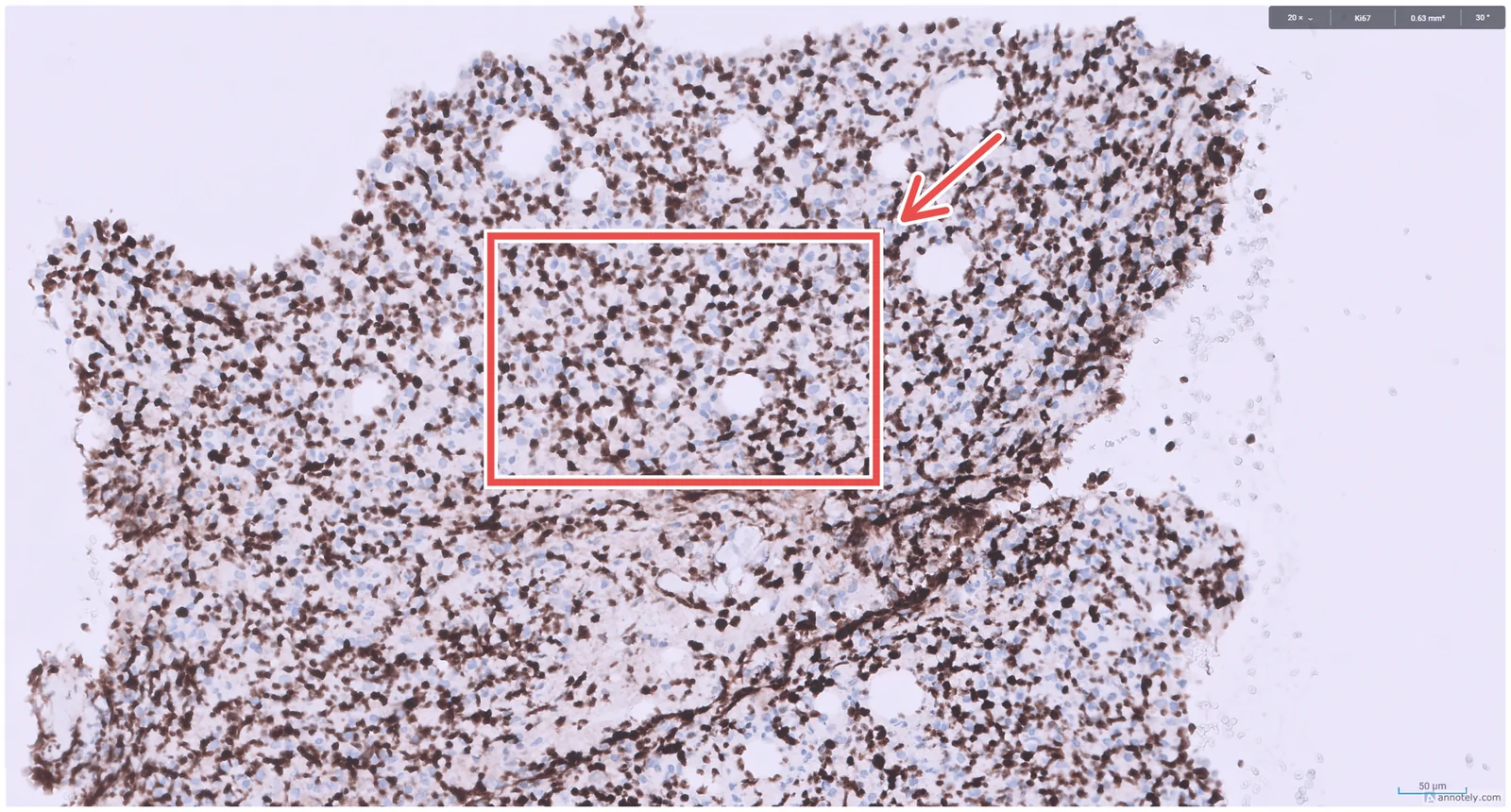
Ki‑67 immunohistochemical staining of bone marrow trephine biopsy. Ki‑67 immunohistochemistry showing nuclear positivity in approximately 70% of neoplastic B cells, consistent with a high proliferative index.

The patient was transferred to haematology and commenced on R-mini-CHOP (rituximab, cyclophosphamide, doxorubicin, vincristine, and prednisolone). Following the first cycle, rapid clinical and biochemical improvement was observed, including the resolution of hyperlactatemia.

MRCP demonstrated diffuse, heterogeneous signal alterations throughout the hepatic parenchyma, suggestive of reactive hepatitis versus congestive hepatopathy, without biliary tree dilatation or cholelithiasis. In the clinical context, these findings were interpreted as HLH-associated reactive hepatic changes, with possible contribution from hepatic lymphoma infiltration, rather than as a separate diagnosis of hepatitis; no specific treatment for hepatitis was initiated (Figure 7). Dedicated pelvic MRI demonstrated diffuse, relatively symmetrical marrow signal abnormalities, reported as favouring red marrow conversion rather than definite marrow infiltration. Myeloma was considered but was felt to be less likely. Subsequent bone marrow trephine biopsy established DLBCL and confirmed marrow involvement histologically.

**Figure 7:**
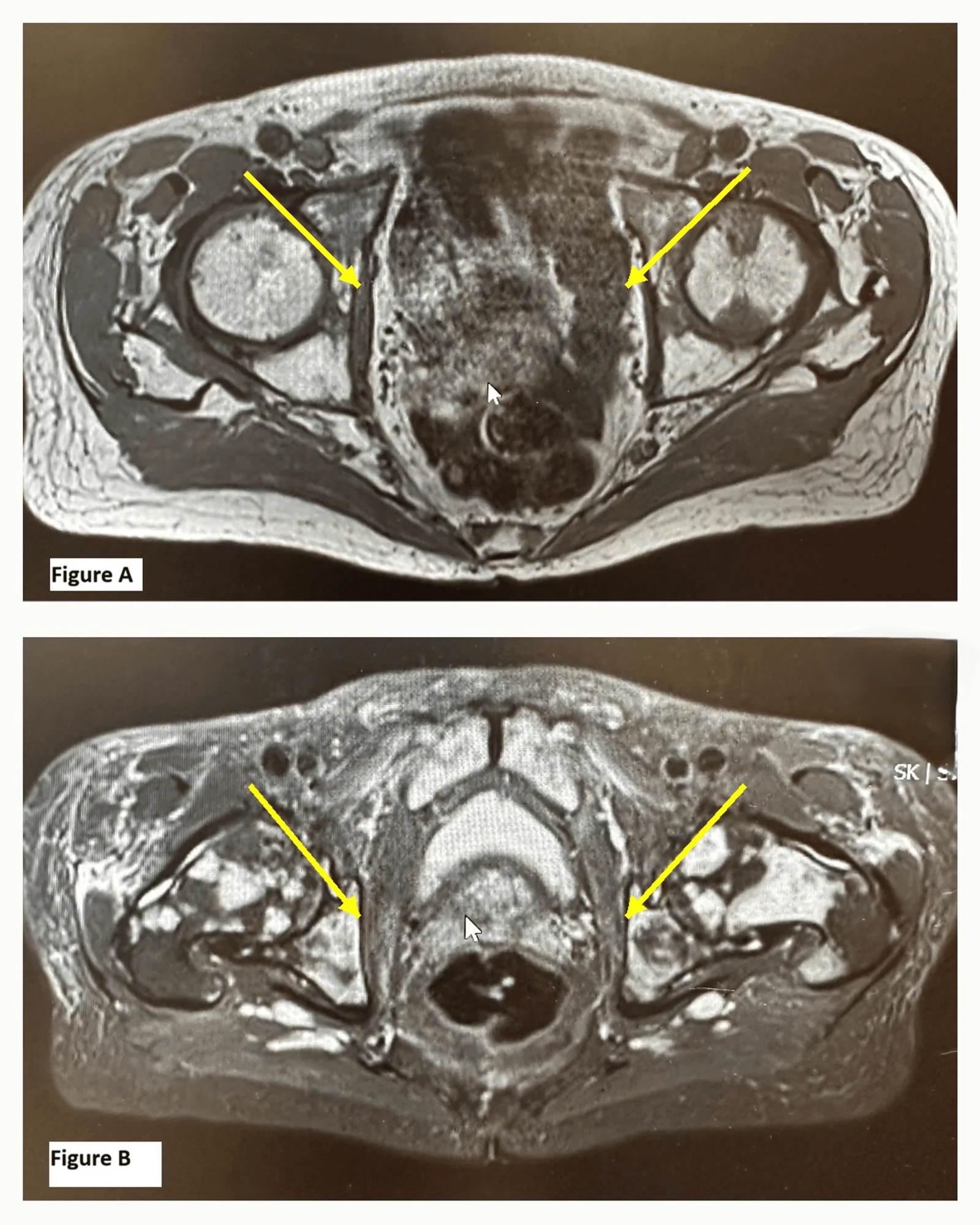
MRI of the pelvis demonstrating diffuse symmetrical bone marrow signal abnormalities. (A) Axial T1-weighted image demonstrating diffuse, relatively symmetrical intermediate T1 marrow signal within the bilateral iliac wings (arrows). (B) Corresponding fluid-sensitive/STIR image demonstrating increased marrow signal within the bilateral iliac wings (arrows). The symmetrical and uniform distribution, with the relative preservation of normal fatty marrow in the femoral diaphyses, was reported as favouring red marrow conversion. STIR: short tau inversion recovery

Following regional lymphoma multidisciplinary team review, the disease was staged as advanced DLBCL with bone marrow and suspected hepatic involvement from MRCP findings and liver dysfunction (stage IV high grade). Interim PET-CT performed after two cycles of R-mini-CHOP demonstrated a complete metabolic response with the normalisation of liver functions.

Treatment response and follow-up

Following the initiation of R-mini-CHOP, the patient's full blood count and liver function tests normalised, with the complete resolution of hyperlactataemia (Table 7).

**Table 7:** Serial haematological, biochemical and HLH‑associated parameters before and during treatment. R-mini-CHOP, reduced-dose rituximab, cyclophosphamide, doxorubicin, vincristine and prednisone/prednisolone; LDH, lactate dehydrogenase; HLH, haemophagocytic lymphohistiocytosis; AST, aspartate aminotransferase; ALT, alanine aminotransferase; ALP, alkaline phosphatase

Parameters	Worse values pretreatment (day 5 of admission)	Day 3 of steroid/anakinra (day 8 of admission)	After first cycle of R-mini-CHOP (day 14 of admission)	After second cycle of R-mini-CHOP (six weeks after initial presentation)	Normal values
Haematological					
Haemoglobin	90	107	82	130	130-170 g/L
White cell count	1.87	6.45	7.75	5.41	3.7-11 10^9^/L
Platelet	14	44	338	185	150-400 10^9^/L
Lymphocyte	0.38	0.65	0.40	1.03	0.9-4.0 10^9^/L
Neutrophils	1.42	4.93	6.58	3.50	1.7-7.5 10^9^/L
Biochemical					
AST	320	130	129	-	<50 IU/L
ALT	272	68	152	14	<50 IU/L
ALP	1091	420	226	97	30-130 IU/L
Bilirubin	55	28	22	1-3	0-21 µmol/L
LDH	4162	-	1758	-	208-378 IU/L
Lactate	14.3	8.1	-	1.1	0.6-2.0 mmol/L
HLH associated					
Ferritin	10866	1539	1092	-	30-300 µg/L
Triglyceride	8.60	6.79	3.58	1.32	<1.7 mmol/L
Fibrinogen	1.2	1.4	1.6	-	2.3-4.7 g/L

His Eastern Cooperative Oncology Group (ECOG) performance status improved from 4 to 1. An interim PET-CT performed after two cycles of R-mini-CHOP demonstrated a complete metabolic response. He continues to receive R-mini-CHOP, with a planned total of five cycles administered at 21-day intervals, with ongoing clinical and laboratory monitoring; further imaging will be performed as clinically indicated.

## Discussion

HLH is a hyperinflammatory syndrome driven by the dysregulated activation of T cells and myeloid cells, resulting in the excessive production of pro-inflammatory cytokines, including interferon-gamma (IFN-γ), interleukin-6 (IL-6) and tumour necrosis factor-alpha (TNF-α) [7]. Haematological malignancies are an important trigger of secondary HLH in adults, with lymphoma representing a substantial proportion of malignancy-associated cases [8]. In this case, HLH was the initial clinical manifestation of previously undiagnosed diffuse large B-cell lymphoma (DLBCL). The absence of clinically apparent lymphadenopathy, hepatomegaly or splenomegaly, together with predominantly nonspecific constitutional symptoms and biochemical abnormalities, made the identification of the underlying lymphoma challenging.

Diagnosing malignancy-associated HLH in adults can be difficult because several clinical and laboratory features overlap with those of the underlying malignancy, including fever, cytopenias, hyperferritinaemia, elevated lactate dehydrogenase and liver dysfunction [9]. HLH is associated with substantial morbidity and mortality, particularly when diagnosis and treatment are delayed. Despite treatment, overall one-year survival has been reported at approximately 50% [10]. The early recognition of HLH, identification of the underlying trigger and prompt initiation of appropriate treatment are therefore important. The HScore can support the assessment of suspected HLH in adults but should be interpreted alongside the overall clinical picture [4,11]. In this case, the diagnosis of HLH was supported by the evolving clinical and biochemical picture and a high HScore. However, identifying the underlying trigger remained challenging, particularly given the concurrent consideration of infection, autoimmune disease and malignancy. As cytopenias and organ dysfunction worsened, these potential secondary causes were investigated in parallel. Given the suspected secondary HLH, worsening cytopenias, clinical deterioration and absence of an identified trigger, haematology recommended a bone marrow trephine biopsy. This subsequently confirmed DLBCL and established the underlying cause of the lymphoma-associated HLH. Importantly, the decision to perform the bone marrow examination was based on the overall clinical picture and was not driven by the hyperlactataemia.

Another notable feature was the persistent hyperlactataemia despite fluid resuscitation and haemodynamic stabilisation, with no identifiable infective source or evidence of ongoing tissue hypoperfusion. This raised the consideration of a type B mechanism. One potential explanation is the Warburg effect, whereby malignant cells preferentially utilise aerobic glycolysis over oxidative phosphorylation despite adequate oxygen availability, resulting in increased lactate production [12]. In this patient, the persistent hyperlactataemia may have reflected a combination of increased lactate production associated with the underlying lymphoma and impaired hepatic clearance in the context of cholestatic liver dysfunction. However, the precise mechanism cannot be established from this case. The persistence of hyperlactataemia despite the correction of apparent type A causes, together with its subsequent resolution following lymphoma-directed treatment, suggests an association with the underlying disease but is not specific for malignancy. Thus, hyperlactataemia should be regarded as an additional clinical feature rather than a diagnostic marker of lymphoma or HLH.

Cholestatic liver dysfunction has a broad differential diagnosis, including biliary obstruction, hepatic infiltration, infection and systemic inflammation. HLH itself can cause hepatic injury through cytokine-mediated inflammation and lymphohistiocytic infiltration, resulting in abnormalities ranging from transaminitis and cholestasis to severe hepatic dysfunction. In this case, there was no evidence of biliary obstruction on imaging, and viral investigations were negative. CT of the abdomen and pelvis and subsequent MRCP did not identify a clear obstructive cause, while multidisciplinary discussion raised the possibility of hepatic involvement by lymphoma. The cholestatic liver dysfunction was therefore likely multifactorial, with systemic inflammation related to HLH and possible hepatic involvement by the underlying lymphoma both contributing.

The management of HLH requires the control of the hyperinflammatory response while simultaneously addressing the underlying trigger. In this case, corticosteroids and anakinra were initiated because of the patient's clinical deterioration and high-risk features of secondary HLH, while investigations to identify the underlying cause continued in parallel. These treatments produced limited clinical and biochemical improvement, with persistent cytopenias, liver dysfunction and hyperlactataemia. Although immunomodulatory therapy can help control the hyperinflammatory response, outcomes in malignancy-associated HLH remain closely linked to the control of the underlying malignancy [8]. Definitive management therefore requires the treatment of both the hyperinflammatory syndrome and its underlying trigger. Following the initiation of R-mini-CHOP, there was marked clinical and biochemical improvement, with lactate falling to 1.1 mmol/L after treatment. After two cycles, the patient's Eastern Cooperative Oncology Group (ECOG) performance status improved from 4 at presentation to 1, and an interim PET-CT demonstrated a complete metabolic response. The concurrent resolution of the hyperlactataemia and cholestatic liver dysfunction following lymphoma-directed treatment further supports lymphoma as an important contributor to these abnormalities, although it does not establish a single underlying mechanism.

This case highlights that occult lymphoma may present with secondary HLH without palpable lymphadenopathy, organomegaly or other classical lymphoma features. Persistent hyperlactataemia and cholestatic liver dysfunction were distinctive features in this patient and, when persistent despite the correction of apparent type A causes, represented additional clinical features that prompted the consideration of an underlying malignancy. However, these findings are not intended to define a general diagnostic pathway. In adults with suspected HLH, systematic evaluation for secondary triggers and timely bone marrow examination should be guided by the overall clinical presentation, including worsening cytopenias, rather than by lactate levels alone.

## Conclusions

Malignancy-associated HLH may be the first manifestation of an otherwise occult haematological malignancy. In this case, unexplained hyperlactataemia prompted evaluation for potential type A causes and, when it persisted despite the correction of these potential causes, raised the consideration of a type B mechanism and an alternative underlying process. In the context of suspected HLH, worsening cytopenias, progressive organ dysfunction and unrevealing conventional imaging, further investigation ultimately established previously occult DLBCL. This case highlights that an unexplained persistent lactate elevation that remains unresolved after the correction of potential type A causes may provide an additional clue to an underlying malignancy in the appropriate clinical context. However, it is not a specific diagnostic marker. Increased lactate production related to lymphoma metabolism may have contributed, but the available data do not establish a specific biochemical mechanism, and impaired hepatic clearance and systemic inflammation may also have played a role. The marked clinical and biochemical response following lymphoma-directed therapy reinforces the importance of identifying and treating the underlying driver of secondary HLH.
